# PixRevive: Latent Feature Diffusion Model for Compressed Video Quality Enhancement

**DOI:** 10.3390/s24061907

**Published:** 2024-03-16

**Authors:** Weiran Wang, Minge Jing, Yibo Fan, Wei Weng

**Affiliations:** 1 School of Microelectronics, Fudan University, Shanghai 200433, China; wrwang0922@gmail.com (W.W.); fanyibo@fudan.edu.cn (Y.F.); 2 Department of Liberal Arts and Science, Kanazawa University, Ishikawa 920-1192, Japan; weng@staff.kanazawa-u.ac.jp

**Keywords:** compressed video restoration, diffusion model, rich detail information, group-wise domain fusion

## Abstract

In recent years, the rapid prevalence of high-definition video in Internet of Things (IoT) systems has been directly facilitated by advances in imaging sensor technology. To adapt to limited uplink bandwidth, most media platforms opt to compress videos to bitrate streams for transmission. However, this compression often leads to significant texture loss and artifacts, which severely degrade the Quality of Experience (QoE). We propose a latent feature diffusion model (LFDM) for compressed video quality enhancement, which comprises a compact edge latent feature prior network (ELPN) and a conditional noise prediction network (CNPN). Specifically, we first pre-train ELPNet to construct a latent feature space that captures rich detail information for representing sharpness latent variables. Second, we incorporate these latent variables into the prediction network to iteratively guide the generation direction, thus resolving the problem that the direct application of diffusion models to temporal prediction disrupts inter-frame dependencies, thereby completing the modeling of temporal correlations. Lastly, we innovatively develop a Grouped Domain Fusion module that effectively addresses the challenges of diffusion distortion caused by naive cross-domain information fusion. Comparative experiments on the MFQEv2 benchmark validate our algorithm’s superior performance in terms of both objective and subjective metrics. By integrating with codecs and image sensors, our method can provide higher video quality.

## 1. Introduction

The thriving development of Internet of Things (IoT) technologies has led to an explosion in video data traffic. However, the massive costs of data storage and limited upload bandwidth pose obstacles to the continuous transmission of high-quality videos. To tackle this challenge, classic video coding standards have emerged, including H.263 [1], H.264/AVC [2], and H.265/HEVC [3]. These schemes leverage the spatial and temporal redundancies in video content to enable efficient transmission and storage through lossy compression. Meanwhile, breakthroughs in image sensor technologies have steadily improved video resolution, dynamic range, and denoising capabilities. This provides superior initial conditions for compression coding and richer quality clues for subsequent video refinement and restoration algorithms. However, inevitable bitrate reduction introduces multifaceted data loss and compression artifacts like blurring, blockiness, and edge fluctuations [4,5]. Such distortions fail to satisfy the requisite user experience quality (QoE) [6,7]. Additionally, disruption and damage introduced in frame coding adversely affect downstream computer vision tasks reliant on video content like scene analysis and object tracking, thus compromising visual fidelity. Therefore, developing powerful compressed video restoration algorithms to rectify compression-induced reductions in image quality is imperative.

Earlier traditional methods typically optimize transform coefficients based on specific compression standards [8,9]. Such codec-dependent approaches struggle to generalize across standards. In contrast, CNN-based methods, like QE-CNN [10], MFQEv2 [11], STDF [5], and RFDA [12], demonstrate superior performance on video enhancement tasks. With the advent of neural network architectures like Vision Transformers [13,14], learning-based video restoration techniques have also made significant strides. State-of-the-art designs such as STCF [15] and TVQE [16] prove effective for restoration. Beyond task-specific solutions, recent research has also established unified frameworks, like BasicVSR [17] and BasicVSR++ [18], to address compression artifacts. However, the limitations imposed by these methods impede their performance, making it challenging to effectively address highly uncertain issues, such as images that are severely damaged or have significant information loss. It is difficult to accurately infer the Possibility distribution of missing parts from the remaining valid pixels. Therefore, tighter integration of sensor technologies and video codecs to generate outstanding high-quality video remains key for advancing compressed video perceptual quality enhancement algorithms.

To address the aforementioned challenges, we intend to utilize cutting-edge conditional generation modeling (diffusion probability model) [19,20] as the foundation. By leveraging advanced sensor imaging systems and robust generative architectures with strong representation and generalization capabilities, we aim to progressively enhance the quality of data during the reconstruction process, thereby generating more intricate and lifelike images. This approach allows the model to focus on detail recovery incrementally, rather than attempting to solve the entire complex problem at once, ultimately improving the efficiency of video restoration. However, without careful guidance, directly applying the diffusion model to video damage repair may disrupt inter-frame dependencies and inevitably lead to detail distortion. To surmount these limitations, we propose a novel synergistic framework between denoising diffusion and CNNs to ameliorate compression video impairments. Our model first extracts edge information from video frames through the ELPNet based on discrete wavelet transform, enabling more targeted and higher-quality reconstruction of high-frequency components. This constructs a pseudo ground-truth feature space guiding the diffusion model’s denoising process. Finally, the outputs are fused together. Through this collaborative framework, highly correlated information complements each other to effectively restore low-quality video, achieving state-of-the-art restoration quality on the MFQEv2 dataset compared to previous approaches.

Our contributions are summarized as follows:

1. We propose the first diffusion-model-based video compression restoration network, surpassing the performance limitations of previous neural network methods.
2. We design a frequency-domain filling block (FFB), the core idea of which is leveraging the multi-resolution frequency-domain features provided by wavelet transforms to guide detail restoration. It provides more high-frequency knowledge to reconstruct sharp texture details.
3. Theoretical analysis reveals domain discrepancies between diffusion models and deep convolutional networks. Direct latent feature fusion may exacerbate these gaps, inducing distortions. To mitigate this, we design a simple yet effective group-wise domain fusion module.
4. Extensive experiments and ablation studies validate the superior performance of our proposed technique.

## 2. Related Work

### 2.1. Compressed Image/Video Restoration

Inspired by the success of deep learning, a multitude of recent works [21,22,23,24,25,26,27,28] have demonstrated that convolutional neural networks (CNNs) exhibit superior performance in enhancing image and video compression quality. The ARCNN designed by Dong et al. [22] pioneers the leverage of CNNs to mitigate artifacts introduced by JPEG encoding. Owing to its robustness, DnCNN [23] is frequently employed as the benchmark for image restoration, including denoising and artifact reduction. QE-CNN [10] utilizes two models to reduce distortions for I frames and P/B frames. MFQEv2 [11] utilizes motion compensation between two adjacent peak quality frames extracted by optical flow estimation to enhance low-quality frames. Additionally, to effectively process motion relations, STDF [5] proposes a spatiotemporal deformable fusion scheme to aggregate temporal information to eliminate unpleasant distortions. RFDA [12] further refines STDF through recursive fusion and deformable spatiotemporal attention modules to simulate long-range motion compensation. To enhance perceptual quality, a new generative adversarial network named MW-GAN+ [29] leverages multi-level wavelet packet transform (WPT) to recover high-frequency details and fine-grained textures. Recently, researchers have introduced Transformer-based frameworks into the field of video compression restoration and achieved promising results. Zhang et al. [15] designed a parallel structure combining Swin Transformer and CNN, which integrates motion compensation and global context information. Another work, TVQE [16], designed novel modules that are capable of not only learning local and global features for correlational modeling but also aggregating inter-frame information. These methods can effectively restore the artifacts caused by video compression. However, these methods falter in reconstructing high-frequency details, especially along image edges. Additionally, over-reliance on intrinsic learning patterns during training hampers texture expressiveness, yielding blurry, smoothed outputs, thus rendering the restoration work unsatisfactory.

### 2.2. Diffusion Models

Diffusion-based [30] generative models have recently regained widespread attention. This class of models sequentially perturbs data samples by introducing additive noise to simplify them into elementary distributions (e.g., Gaussian), then reverses the process, and learns to recover the latent variables in the simple distribution back to data in the complex distribution by optimizing a variational lower bound of the likelihood function, using parameterized Markov chains. Subsequently, these models gradually denoise samples from the noisy distribution via Langevin dynamics [31], yielding target samples from the data distribution.

Recently, DDPM [32] has shown state-of-the-art performance across various tasks, including image super-resolution [33,34], restoration [35,36], and translation tasks (restoration, colorization, etc.) [37,38]. Additionally, the learned feature representations from diffusion models also prove very useful for discriminative tasks, including image classification [39], segmentation [40,41], and object detection [42]. Diffusion models have been extensively used for sample generation owing to the high quality and diversity of their generated samples. With the continuous advancement of diffusion models across domains, they have surpassed the long-standing dominance of GANs in image generation. However, intrinsic defects persist for utilizing diffusion models in video restoration. Specifically, we have empirically shown, through experiments, that merely applying diffusion models fails at temporal modeling, contrarily deteriorating performance. Hence, our work ingeniously overcomes the innate deficiencies of diffusion models in inter-frame modeling through innovative architectural designs.

### 2.3. Neural Network Combined with Diffusion Model

To better enhance the image restoration capability of diffusion models, existing works [43,44] incorporate latent features from conditional neural networks into training diffusion models. Specifically, the method extracts integrated features from low-resolution images through a neural network for conditioning to guide image generation. Then, the neural network features are simply linearly combined with the probability distribution features from the diffusion model; while moderately improving restoration on specific domains, there are some limitations: (1) the weak detail restoration capabilities; (2) disregarding domain discrepancies and simply conducting linear fusion lead to unsatisfactory detail effects or even distortions; and (3) the fusion mainly aims to improve restoration on specialized domains rather than generalizing to common visible light images. In contrast, our method has three main advantages: (1) Our guiding network leverages discrete wavelet transforms to obtain richer texture details, abstracted into the latent space for enhancing detail restoration and generation capacity. We then integrate this wavelet-enhanced network with the diffusion model for targeted performance gains. (2) We devise a simple yet effective patch-wise domain matching module to bridge domain gaps for seamless fusion, alongside an efficient fusion mechanism. (3) We have extended our model to common visible light domains and achieved state-of-the-art results.

## 3. Preliminaries: Diffusion

**Figure 1:**
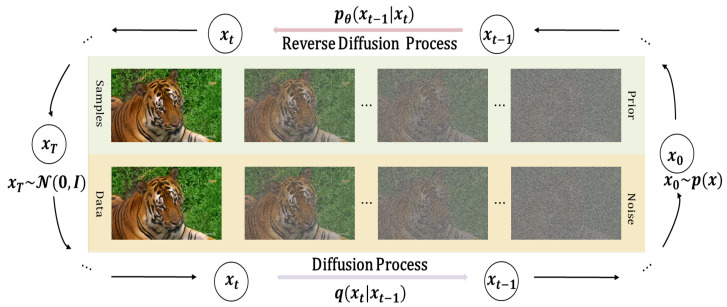
The diffusion process and inverse diffusion process of diffusion models for compressed video frame restoration.

In this paper, we adopt diffusion models to generate accurate restorations for compressed damaged video frames. This is achieved by learning Markov chains that progressively convert the Gaussian noise distribution to the trained model’s data distribution. The process comprises two key phases: forward diffusion and reverse diffusion. As illustrated in Figure 1, given the true data distribution $x_{0}∼p\left(x\right)$, the forward diffusion process injects Gaussian noise over T timesteps to incrementally corrupt the distribution. This yields a series of noisy samples, parametrized by the variance schedule ($\beta_{1},\beta_{2},\cdots \;,\beta_{t})$. Noise samples denote latent variables sharing the original data dimension. Each iteration of the forward process, transforming $x_{0}$ into $x_{T}∼N\left(0,1\right)$, can be described as:

(1) $$p\left(x_{t}|x_{t-1}\right)=N\left(x_{t};\sqrt{1-\beta_{t}}x_{t-1},\beta_{t}I\right).$$

For ease of calculation and formula representation, let $\alpha_{t}=1-\beta_{t},{\bar{\alpha }}_{t}=\prod_{i=0}^{t}\alpha_{i}$; Equation (1) can be further reduced to:

(2) $$p\left(x_{t}|x_{0}\right)=N\left(x_{t};\sqrt{{\bar{\alpha }}_{t}}x_{0},\left(1-\sqrt{{\bar{\alpha }}_{t}}\right)I\right).$$

This suggests that the data distribution $p(x_{t}|x_{0})$ can be computed directly from Equation (2) for any moment *t* without iteration. As *t* increases, the fraction of the introduced noise escalates, while that of the original data $x_{0}$ diminishes. When Gaussian noise dominates, the distribution of $p(x_{t}|x_{0})$ converges to the Gaussian distribution N(0,I), indicating the completion of the forward diffusion phase where structural information corrodes.

The learning of diffusion models is achieved by reversing the forward process defined in Equation (1) to construct a reverse Markov chain. Specifically, define a joint distribution $p_{\theta }\left(x_{0},\cdots \;,x_{T}\right)$ controlled by θ, and then construct a reverse process based on this joint distribution, that is, starting from the standard normal distribution $p\left(x_{T}\right)=N\left(x_{T};0,I\right)$, perform Gaussian denoising step by step until approximating the true data distribution. The formulas are as follows:

(3) $$q_{\theta }\left(x_{0},\ldots ,x_{T-1}|x_{T}\right):=\prod_{t-1}^{T}\;q_{\theta }\left(x_{t-1}|x_{t}\right),$$

(4) $$q_{\theta }\left(x_{t-1}|x_{t}\right):=N\left(x_{t-1};\mu_{\theta }\left(x_{t},t\right),\sigma_{\theta }{\left(x_{t},t\right)}^{2}I\right).$$

The parameters involved in the backward process, such as $\mu_{\theta },\sigma_{\theta }\;$, represent the mean and variance of the Gaussian distribution, respectively, which are estimated by a neural network. In addition, the sequence of variances $\beta_{t}$ mentioned in the forward process can participate in joint model learning or remain unchanged.

In the training phase, we construct an upper bound on the negative log-likelihood by adding a non-negative KL dispersion term to the negative log-likelihood function $-logp_{\theta }\left(x_{0}\right)$ of the target data distribution $p_{\theta }\left(x_{0}\right)$, denoted as Equation (5), and the specific expansion can be expanded into [32].

(5) $$\begin{array}{cc} \;-\log p_{\theta }\left(x\right) & \leq -\log p_{\theta }\left(x_{0}\right)+D_{KL}\left[q\left(x_{1:T}|x_{0}\right)∥p_{\theta }\left(x_{1:T}|x_{0}\right)\right]\\ E_{q\left(x_{0}\right)}\left[-\log p_{\theta }\left(x_{0}\right)\right] & \leq E_{q}[\underset{L_{T}}{\underset{︸}{D_{\mathrm{KL}}\left(q\left(x_{T}|x_{0}\right)∥p\left(x_{T}\right)\right)}}\underset{L_{0}}{\underset{︸}{-\log p_{0}\left(x_{0}|x_{1}\right)}}\\ \; & +\sum_{t>1}\underset{L_{T-1}}{\underset{︸}{D_{\mathrm{KL}}\left(q\left(x_{t-1}|x_{t},x_{0}\right)∥p_{\theta }\left(x_{t-1}|x_{t}\right)\right)}}], \end{array}$$

In the $L_{T-1}$ term in the above formula, the KL divergence of the two Gaussian distributions $p_{\theta }\left(x_{t-1}|x_{t}\right)$ and $q(x_{t-1}|x_{t},x_{0})$ is calculated; the latter is based on the original data $X_{0}$. The posterior distribution of the true unknown generation process is inferred from the global perspective of the entire diffusion model. The specific expression is as follows:

(6) $$q\left(x_{t-1}|x_{t},x_{0}\right)=N\left(x_{t-1};{\tilde{\mu }}_{t}\left(x_{t},x_{0}\right),{\tilde{\beta }}_{t}I\right),$$

where mean $\mu_{t}\left(x_{t},x_{0}\right)=\frac{1}{\sqrt{\alpha_{t}}}\left(x_{t}-\epsilon \frac{1-\alpha_{t}}{\sqrt{1-{\bar{\alpha }}_{t}}}\right)$, variance ${\tilde{\beta }}_{t}=\frac{1-{\bar{\alpha }}_{t-1}}{1-{\bar{\alpha }}_{t}}\beta_{t}$, and ϵ represents the noise in $x_{t}$, which is the only uncertain variable in the reverse process. The diffusion model uses a denoising network $\epsilon_{\theta }\left(x_{t},t\right)$ to estimate ϵ. Finally, based on the description in [32], we perform the parameter optimization of the network by means of Equation (7).

(7) $$\nabla_{\theta }{∥\epsilon -\epsilon_{\theta }\left(\sqrt{{\bar{\alpha }}_{t}}x_{0}+\epsilon \sqrt{1-{\bar{\alpha }}_{t}},t\right)∥}_{2}^{2}.$$

## 4. Approach

**Figure 2:**
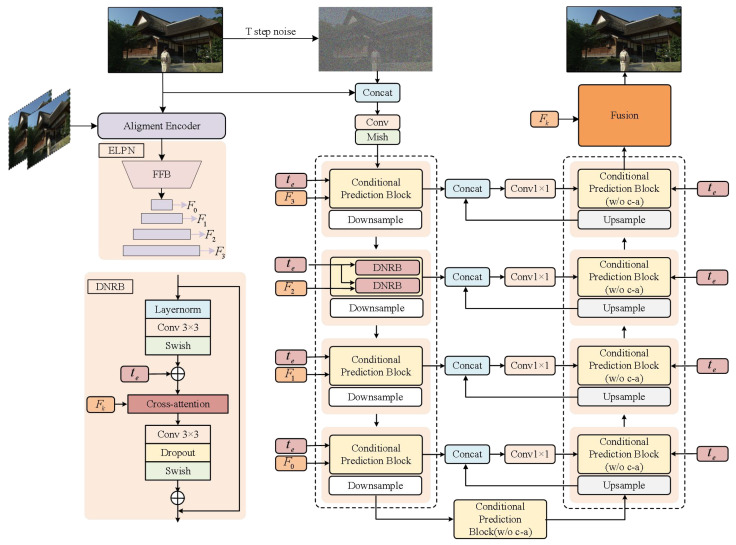
The overall architecture of the proposed LFDM. First, the current frame and neighboring frames are fed into the ELPN for pre-training. Second, the ELPN extracts the prior latent features and feeds them to the CNPN to direct its generation process. The details of the CNPN are illustrated in the figure. Finally, feature information from different domains is consolidated via “fusion”, comprehensively elaborated on in Section 4.3. Here, t∼Uniform{1,…,T} and transformed into $t_{e}$ through an MLP; FFB represents the frequency-domain filling block; (w/o c-a) denotes without cross-attention.

Given a compressed low-quality video sequence, $V_{lq}=\left\{X_{k}\in \right.$$\left.R^{C\times H\times W}\right\}$ with K frames, where k∈(1,K). *C*, *H*, and *W* denote the channel, height, and width of each frame, respectively. As shown in Figure 2, we demonstrate the overall pipeline of the Latent Feature Diffusion Model (LFDM). In our methodology, we feed the current frame into ELPN and additionally introduce adjacent frames to enhance the richness of the original input information, which enables the network to construct a more coherent spatiotemporal representation, thus preserving inter-frame dependencies. The corresponding reference frame input is $X_{f}=\left\{X_{k-1},X_{k},X_{k+1}\right\}$. When enhancing it into a high-quality frame, we extract and store the mapped features as a pseudo ground-truth feature bank to provide more accurate conditional features for reverse diffusion. This allows the diffusion model to probe a solution space akin yet not identical to the conditional features, chasing improved outcomes while retaining correlation with the multi-frame data. We use Equation (2) to convert $X_{k}$ into $P(X_{t}|X_{k})$ as the input for the diffusion model. Finally, fusing its output with the repository features produces the optimal result. Overall, the enhanced frame ${\hat{Y}}_{t}$ of the compressed frame $X_{k}$ is generated as:

(8) $$\begin{array}{cc} F_{k} & =F_{\mathrm{con}}\left(X_{f}\right),\;\;k\in \left\{0,1,2,3\right\},\\ D_{i}^{P} & =P(X_{t}|X_{k}),\\ D_{o}^{P} & =\mathrm{Diff}\left(D_{i}^{P},F_{k}\right),\;\;k\in \left\{0,1,2,3,\right\},\\ {\hat{Y}}_{t} & =\mathrm{Fusion}(D_{o}^{P},F_{k}), \end{array}$$

where $F_{\mathrm{con}}\left(\cdot \right)$ denotes the decoder of the ELPNet, $\left\{F_{k}|k\in 0,1,2,3\right\}$ represents decoding-end features of varying sizes extracted from the ELPNet, Diff(·) refers to the diffusion model’s conditional denoising network, and fusion signifies the final module fusing information across domains. This effectively mitigates deficiencies induced by directly fusing cross-domain features, thereby unleashing the potential of heterogeneous information to better achieve the target task. The details of ELPNet, diffusion, and fusion will be elaborated in Section 4.1, Section 4.2, and Section 4.3, respectively.

### 4.1. ELPNet

Before introducing the ELPNet, we first present a spatiotemporal alignment module [45] that harnesses optical flow estimation (OFE) to compute forward and backward flows between adjacent frames. These optical flows then warp the input frames temporally, which is vital to leverage useful information from neighboring frames for restoring the target.

**Figure 3:**
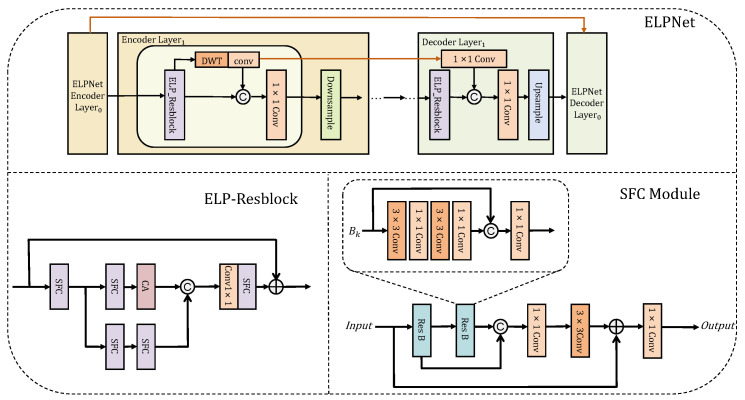
The overall structure of our proposed ELPNet. DWT refers to the Discrete Wavelet Transform and CA denotes the Channel Attention mechanism.

Our CNN branch, namely, ELPNet (Figure 3), aims to directly learn the mapping from damaged to pristine images. Its encoded integrated features serve as conditioning to guide diffusion model generation. To achieve this, we adopt the same architecture as the diffusion model’s denoising network for constructing the ELPNet. By conducting feature extraction through ELP-Resblock (structure in Figure 3, left), which blends frequency-domain information using Discrete Wavelet Transforms (DWTs), we can retain more texture details during restoration while forcing the network to learn both high and low frequencies.

Specifically, a fixed-parameter low-pass filter ($L_{FF}$) and high-pass filters ($H_{FF1}$, $H_{FF2}$, and $H_{FF3}$) perform stride 2 convolution calculations to decompose images or feature maps into four sub-bands ($X_{LF}$, $X_{HF1}$, $X_{HF2}$, $X_{HF3}$). We denote $X_{LF}$ as $\left(L_{FF}⊛X\right){↓}_{2}$, which represents the convolutional computation, where ${↓}_{2}$ indicates a 2x scaling factor. We embed the Haar DWT [46] into our proposed network, $L_{FF}=\left[\begin{array}{c} \begin{array}{cc} 1 & 1\\ 1 & 1 \end{array} \end{array}\right],H_{FF1}=\left[\begin{array}{c} \begin{array}{cc} -1 & -1\\ 1 & 1 \end{array} \end{array}\right],H_{FF2}=\left[\begin{array}{c} \begin{array}{cc} -1 & 1\\ -1 & 1 \end{array} \end{array}\right],H_{FF3}=\left[\begin{array}{c} \begin{array}{cc} 1 & -1\\ -1 & 1 \end{array} \end{array}\right]$. Then, the value at the (i,j)-th position of $X_{LF}$ after 2D Haar wavelet transformation can be calculated by Equation (9):

(9) $$\begin{array}{cc} X_{LF}\left(i,j\right)= & X(2i-1,2j-1)+X(2i-1,2j)+X(2i,2j-1)+X(2i,2j). \end{array}$$

The expressions for the high-frequency sub-bands are similar to the expression for $X_{LF}$. The integration of low-frequency components as encoding side features with downsampled features provides powerful semantic information and a relatively coarse spatial layout. Furthermore, high-frequency components are integrated into the decoding side region through a multitude of skip connections, guaranteeing the preservation and enhancement of fine image details during the image reconstruction phase. This approach enables our network to not only amalgamate rich information from spatial and frequency domains during the learning process but also enhances its capability to capture high-frequency features like image textures and contours. The experimental results show that the embedding of DWT indeed greatly improves the restoration capability of the network (see Section 5.3 for details).

To ensure the retention of ample texture information in the final restoration results, thereby assisting the diffusion model in recovering intricate and clear details, we apply the following loss function to ELPNet for training, which can be represented as:

(10) $$L=L_{\mathrm{Char}}+\alpha L_{\mathrm{MS}}+\beta L_{\mathrm{Per}},$$

where $L_{\mathrm{Char}}$ refers to Charbonnier loss [47], $L_{\mathrm{MS}}$ refers to MS-SSIM loss [48], and $L_{\mathrm{Per}}$ is perceptual loss [49]. After experimentation, α = 0.2 and β = 0.001 were finally determined as the hyperparameter weights for each loss function part.

### 4.2. Noise Prediction with Modified Conditional Feature

At this stage, we aim to harness diffusion models’ powerful data generation capability for restoring video frames. Initially, ELPNet’s pretrained decoder produces dimension-aligned decoding features as conditioning to guide restoration. This establishes meaningful associations between the target view and the rectified feature view, enhancing the diffusion model’s holistic image understanding to improve detail generation fidelity. An auto-alignment strategy is adopted throughout to ensure alignment between decoded features and corresponding generation content. By effectively utilizing decoded features’ contextual information, this adjusted alignment strategically guides the generation process.

Specifically, the predictor’s main network adopts a U-Net [50] architecture comprising encoder, middle, and decoder steps. The input $D_{i}^{P}$ first undergoes 2D convolutions and Mish activations to extract suitable features. Next, within the Resblocks, cross-attention fuses the pseudo ground-truth features with the denoiser’s intermediates, guiding the network to produce accurate predictions. This is formulated as:

(11) $$\begin{array}{cc} p\left(x_{t-1}|x_{t},F_{k}\right), & =N\left(x_{t-1};\mu \left(x_{t},F_{k}\right),\sigma_{t}^{2}I\right),\\ D_{en0}^{P} & =D_{enc0}\left(F_{k},D_{i}^{P}\right),k=4,\\ D_{en1}^{P} & =D_{enc1}\left(F_{k},D_{en0}^{P}\right),k=3,\\ D_{en2}^{P} & =D_{enc2}\left(F_{k},D_{en1}^{P}\right),k=2,\\ D_{en3}^{P} & =D_{enc3}\left(F_{k},D_{en2}^{P}\right),k=1. \end{array}$$

Formula (11) demonstrates our latent image features guiding diffusion model generation toward high detail retention. Multi-resolution image features ensure the model obtains adequate guidance under varying receptive fields for improved representations. Moreover, our guidance derives from the designed prior frequency-domain blocks, enriching textures and sharpening salient patterns. Consequently, the architecture’s detail restoration and generation capabilities significantly improve. Specifically, time t is sinusoidally position-encoded as $t_{e}$ and embedded via multilayer perceptrons (MLPs) [51]. Every encoder step has two conditional prediction blocks (CPBs) and a downsampling block where 2D convolutions with a stride of 2 are employed to halve the size of the feature map. Each decoder step contains two CPBs without cross-attention and an upsampling block, doubling the size via transposed convolutions. Applying two-dimensional convolution on decoder outputs reconstructs the predicted noise value δϵ to recover $x_{t-1}$ over T iterations, generating the restored frame.

### 4.3. Multi-Scale Group-Wise Information Fusion

Since the output of the conditional neural network belongs to the latent image feature distribution, and the output of the deep diffusion model belongs to the conditional probability distribution, there is a large domain discrepancy between them. If they are directly linearly or nonlinearly combined, the desired performance results cannot be obtained. The existing methods, such as those in [43,52], that fuse convolutional neural networks with diffusion models directly fuse features from the two domains with gaps, which will inevitably lead to image distortions and detail losses. Therefore, how to organically and concisely achieve the fusion of the two has become a universally recognized challenge. This method proposes a simple and innovative solution.

**Figure 4:**
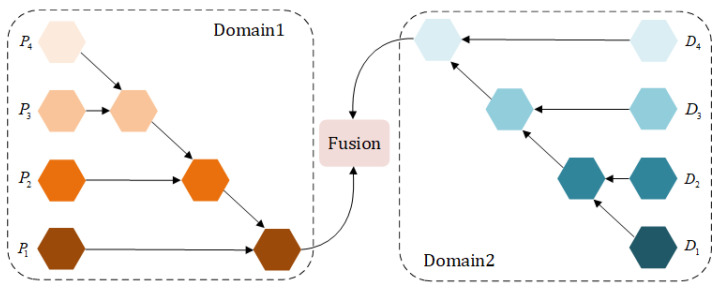
The structure of the fusion module. The left half is the image domain features obtained from the neural network, the right half is the probability distribution features obtained from the diffusion model, and the center represents the fusion of the heterogeneous information.

According to the difference between the two domains, we have designed two different fusion paths and finally set up a reasonable network module to fuse them, which ensures effective alignment of their features. As shown in Figure 4, we use the diffusion denoising network to extract multi-scale features and fuse them with ELPNet’s features. For the denoising backbone of this paper, its extended part contains four convolutional layers, with the output feature size ranging from (8C, H/8, W/8) to (C, H, W). We use a multi-scale feature fusion module to fuse the feature information of the four stages. Eventually, these features are summed and sequentially fed into the fusion head, producing the final result ${\hat{Y}}_{t}\in R^{3\times H\times W}$. Specifically, three dilated convolutions with different dilation rates (r = 1, 2, 4) are applied to map the high-dimensional combination of the two branches to a 3-channel output. Each pixel is acquired by convolutions with 3 × 3, 5 × 5, and 7 × 7 receptive fields, using Leaky ReLU as the activation function.

Using a simple linear weighting method may not result in more enriched semantic representations. The features extracted by different models may overlap and contain redundancies, and directly combining them could exacerbate this issue, ultimately causing a decline in model efficiency. This implicit cross-domain fusion circumvents direct feature interaction across domains; specifically, by introducing an implicit layer, it ensures that the aggregation of information does not hinder the flow of information between different domains. This allows the final features to interact and fuse in a carefully designed common space, enabling information from different domains to complement each other while maintaining their independence, greatly mitigating the negative impacts of mismatch. This strategy helps prevent anomalous uncertain restorations in the outputs. Essentially, this succinct and controllable fusion technique yields more continuous, coherent, and logical restored details. By better achieving our targeted task, it generates more realistic and naturalistic results.

## 5. Experiments

### 5.1. Dataset

We chose to utilize the widely acknowledged MFQEv2 [11] standard dataset within the realm of image and video compression for training our pre-trained models, the ELPNet and the conditional noise prediction network (CNPN). Subsequently, we conducted evaluations to assess the effectiveness of our approach. This dataset encompasses 126 video sequences sourced from Xiph, VQEG, and JCT-VC [53], spanning diverse content and resolutions, establishing it as a robust benchmark for evaluating algorithmic robustness. Adhering to prevailing evaluation standards in this domain, we adhered to a training set–test set ratio of 6:1 for data partitioning. All video sequences underwent compression processing at three different compression rates (QP values of 27, 32, 37, and 42) using HM16.20 and HEVC LowDelay-P (LDP) configurations. Elevated compression rates correspond to more pronounced compression distortions. The utilization of various compression rates enables a comprehensive evaluation of the method’s recovery and generalization capabilities across different levels of compression distortion. In our algorithmic comparative experiments, we conducted an impartial assessment, taking into account the impact of content complexity, resolution, and compression rate on image and video quality.

### 5.2. Experiment Settings

In our research, we developed a model consisting of two key networks: ELPNet, responsible for extracting information from compressed videos to recover corrupted frames, and a conditional noise predictor, a diffusion model network based on the U-Net architecture, for performing the final video frame restoration. Both networks are designed to receive 64 input channels (C = 64). During the training phase of the model, we randomly crop small blocks of 128 × 128 pixels from compressed videos, which serve as training samples to simulate the original data. To enhance the model’s robustness in handling video jitter, we applied a series of data augmentation operations to the training dataset, including random rotation and flipping. We used the Adam optimization algorithm to update the parameters of the conditional noise predictor, where the learning-rate-related hyperparameters $\delta_{1}$ and $\delta_{2}$ were set to 0.9 and 0.999. In the training process of the diffusion model, we empirically set the forward and backward diffusion steps to 1000 steps. Additionally, the selection of noise sequences $\beta_{1},\cdots \;,\beta_{T}$ followed the recommendations in the literature. At the beginning of training, the learning rate was set to $1\times 10^{-4}$ and decreased to one-tenth after completing 70% of the iteration cycles. All experiments were conducted on a high-performance server equipped with an Intel Core i9-13900K CPU, 64 GB of memory, and two NVIDIA ^®^ GeForce RTX 4090 GPUs (NVIDIA, Santa Clara, CA, USA), using PyTorch 2.0.0, Python 3.9, CUDA 11.8, and CuDNN 8.6.0. Building upon the method put forth in [54], this paper implements a repair approach with unconstrained dimensions. In the evaluation process, we used two main performance metrics to quantify the improvement in video quality: Peak Signal-to-Noise Ratio (PSNR) and Structural Similarity Index (SSIM). These standardized metrics allow us to accurately measure and compare the effect of the proposed model on enhancing the quality of video frames.

### 5.3. Comparisons with Previous Algorithms

We presented optimal results on the MFQEv2 dataset, including ARCNN [23], DnCNN [24], MFQEv2 [11], STDF-R3L [5], and RFDA [12]. The results of several of these methods are cited from the relevant literature, and the relevant parameters are strictly configured according to the authors’ recommendations in their publications. Recently the BasicVSR++ [17] method has demonstrated state-of-the-art performance on several video restoration tasks [55]. Considering that the official version of BasicVSR++ was pre-trained and fine-tuned on other datasets, for a fair comparison, we re-trained BasicVSR++ on the MFQEv2 datasets (QP32 and QP37), keeping the same experimental setup as other Baseline methods.

#### 5.3.1. Qualitative Visual Effect Comparison

**Figure 5:**
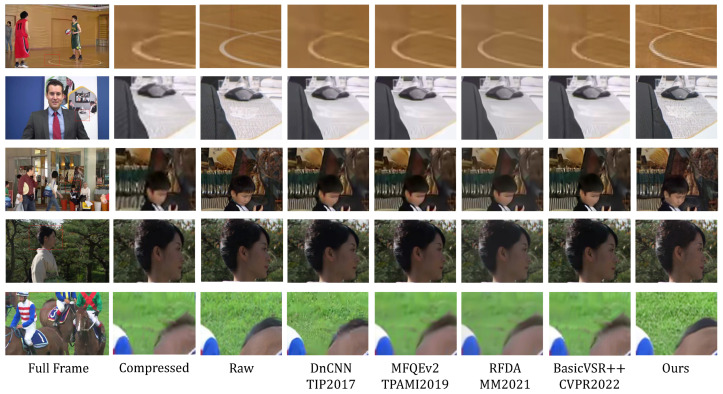
Subjective comparison results between state-of-the-art methods and our proposed method in five video sequences at QP = 37. Test video names (from top to bottom): BasketballPass, Johanny, BQMall, Kimono, and Racehorses. The zoom-in of red box area is shown.

Our method yields visually satisfying results, as depicted in Figure 5, highlighting its exceptional capability to restore intricate details and textures within enhanced frames. In comparison to alternative methods, our restoration outcomes closely align with the ground truth, devoid of issues like excessive smoothing and detail loss. This robustly affirms the effectiveness of our method in rectifying details and texture information in damaged images. Notably, in the BasketballPass sequence, it is evident that contours and object boundary details lost during the compression process are effectively reinstated in our results. The Racehorses sequence similarly showcases this effect, illustrating the preservation of details and textures. The robust capability of our method for detail reconstruction is attributed to the innovative design of the model architecture. The incorporation of detail/texture-sensitive components in the loss function and the integration of a multi-scale sub-network empower the network to adeptly learn how to reconstruct rich and realistic details from contextual information within damaged regions. This presents a robust and effective solution for enhancing the quality of detail and texture restoration in image recovery tasks.

#### 5.3.2. Quality Fluctuation

**Figure 6:**
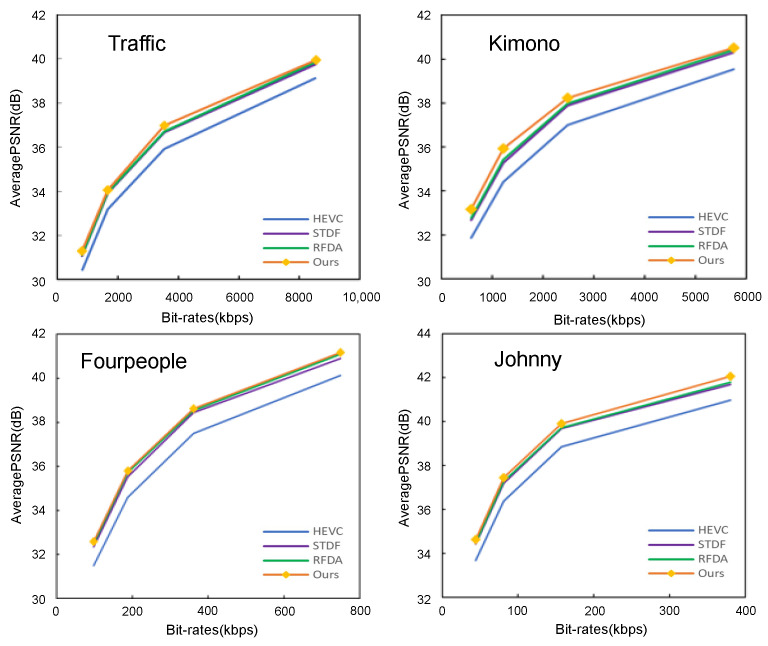
Rate–distortion curves of four test sequences.

Fluctuations in video quality serve as critical evaluation metrics [11]. Random variations in quality can result in significant temporal inconsistencies and a diminished user experience. We utilize Standard Deviation (SD) and Peak–Valley Difference (PVD) [56] to quantify the quality fluctuations for each test sequence. Table 1 presents the average PVD and SD values for different methods across all test sequences. The results indicate that our proposed method exhibits the smallest average PVD and SD. This suggests that, in comparison to other baseline methods, our approach demonstrates smaller quality fluctuations, contributing to a more stable enhancement effect. Furthermore, Figure 6 depicts four PSNR curves for various test sequence groups, representing the original HEVC compressed sequence, RFDA, BasicVSR++, and our method’s processed sequences. It is evident that, when compared to alternative methods, our approach achieves significantly improved performance on compressed frames, demonstrating the lowest fluctuation amplitude.

#### 5.3.3. Rate–Distortion Performance

**Figure 7:**
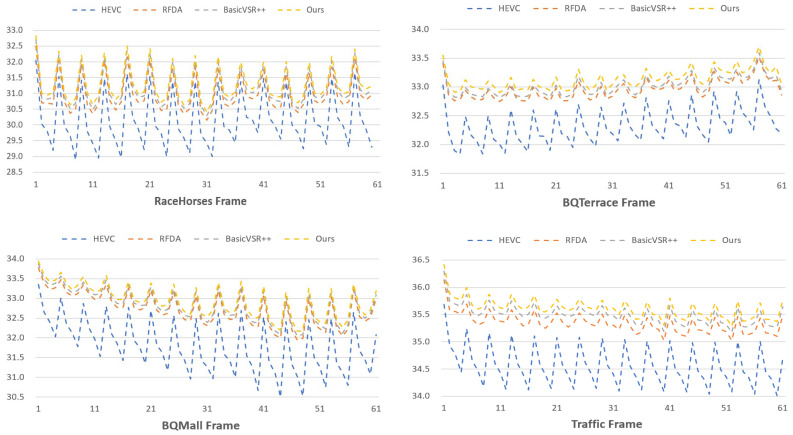
PSNR curves of HEVC, RFDA, BasicVSR++, and ours on four test sequence Cactus at QP = 37.

In comparison to other methods, we conducted a comprehensive evaluation of the rate–distortion performance of our proposed approach. Figure 7 illustrates the rate–distortion curves for our method and other state-of-the-art methods on four selected sequences. The observation reveals that, at similar bit rates, our method consistently attains a higher PSNR compared to other methods, indicating its superior rate–distortion performance.

#### 5.3.4. Overall Performance

Table 2 illustrates the overall improvement of our method in terms of PSNR and SSIM metrics. The results indicate that, regardless of the QP value, our method surpasses other state-of-the-art methods in terms of average metric improvement. For instance, compared to BasicVSR++, we achieve an improvement of 0.13–0.20 dB in PSNR. When contrasted with STCF, our method exhibits a PSNR improvement ranging from 0.02 to 0.06 dB, with a more pronounced enhancement in SSIM. Unlike BasicVSR++ with a bidirectional motion compensation mechanism and STCF’s 7-video-frame restoration approach, our method enhances the target frame by exploring richer texture details and global contextual information through adjacent frame fusion. This is attributed to the targeted design of our diffusion model based on prior latent feature modulation and the group-wise domain fusion module. The extensive experimental results validate the overall superiority of our method in the task of compressed video restoration.

### 5.4. Ablation Study

#### 5.4.1. The effect of ELPNet and fusion in participation

In this section, the results of ablation experiments convincingly demonstrate a significant improvement in the performance of the restoration network when the features from the ELPNet are integrated, as compared to using either the diffusion model alone or the ELPNet in isolation. As shown in Table 3, when the features extracted by the ELPNet are not included, the PSNR and SSIM indices of the diffusion model are noticeably lower. Similarly, when only the ELPNet is utilized for restoration, there is a significant decrease in performance due to the inability to leverage the diffusion model to generate missing image structures. Ultimately, the complete network, after integrating ELPNet features, achieves the optimal improvement in PSNR and SSIM (1.08/1.93). This underscores that the prior latent features extracted by the ELPNet provide crucial guidance for the diffusion model, resulting in the generation of higher-fidelity restoration results through fusion. The synergy between the two components mutually enhances the final image quality. Therefore, incorporating the ELPNet structure in the restoration network is deemed essential, playing an indispensable role in improving restoration effectiveness. The experimental results validate that a single model struggles to achieve a balance between preserving fine details and maintaining overall structural coherence. In this context, feature fusion provides a valuable avenue for complementary enhancement.

**Figure 8:**
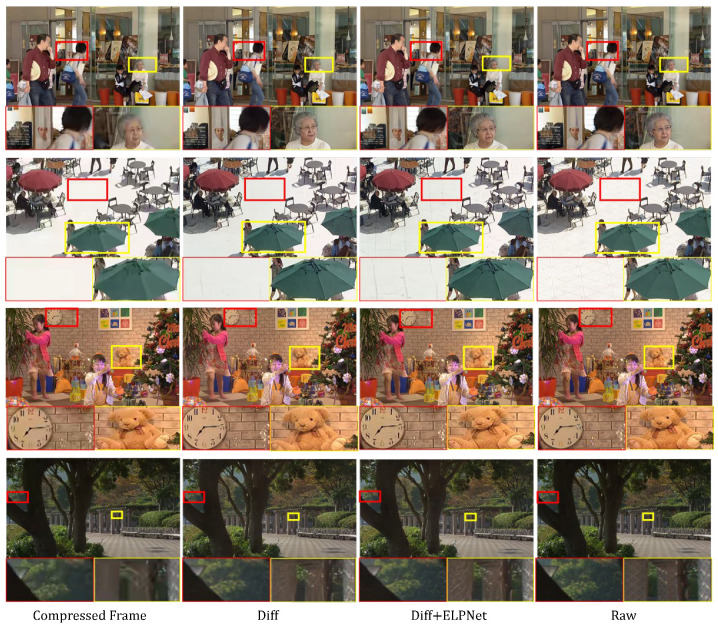
Subjective comparison images depicting the restoration with and without ELPNet intervention. The zoom-in of red box and yellow box area is shown.

Figure 8 depicts subjective comparison images of our method with and without ELPNet involvement in the diffusion model. It is evident that, upon introducing the prior latent features extracted by the ELPNet, the generated results progressively align with the real image, showcasing an enhancement in texture details.

#### 5.4.2. The significance of DWT

To thoroughly substantiate the pivotal role of Discrete Wavelet Transform (DWT) in augmenting image restoration quality, we conducted a comparative analysis of the network’s performance before and after the integration of the DWT module. As depicted in Table 4, the experimental findings distinctly showcase a significant enhancement in various evaluation metrics for ELPNet with DWT, compared to the standard network lacking the DWT module. Notably, the PSNR metric exhibited an increase of 0.08, while the SSIM metric witnessed a noteworthy improvement of 0.27. The significant improvement lies in the substantial increase in SSIM metrics, particularly noticeable in the reconstruction of texture details, highlighting the critical role of frequency-domain information in reinstating high-frequency content in damaged images. This robustly affirms that the DWT module empowers the network to assimilate frequency domain prior knowledge, thereby producing visually richer and more realistic texture effects. Consequently, it can be conclusively stated that the introduction of wavelet transforms is pivotal for elevating the quality of image restoration. The DWT module devised in this study assumes an indispensable role in the reconstruction of details and texture information.

#### 5.4.3. Addition of loss function

In addition, we underscore the importance of the employed loss functions in this study. Upon scrutiny of rows two to four in Table 4, it becomes evident that each loss plays an effective and pivotal role in enhancing both PSNR and SSIM. The Charbonnier loss offers pixel-level supervision, while the perceptual loss guarantees that the output consistently aligns with the ground truth within the deep feature space. Through the comprehensive integration of all losses during the training phase, our model attains optimal performance.

## 6. Conclusions

We propose a novel LFDM approach, completing compressed video damage restoration by designing a neural network combined with sensors and codecs to generate detail-preserving latent features. These judiciously guide the diffusion model to recover fine-grained image information. Specifically, we modulate the diffusion probability distribution by enhancing neural network detail perception using Discrete Wavelet Transforms. Cross-attention is particularly effective for guiding the model’s probability distribution features. Additionally, considering the domain discrepancy between neural networks and diffusion models, our simple yet effective group-wise domain fusion module integrates both to mitigate detail losses and distortions. This substantially boosts model performance. Systematic experiments on public datasets verify our model’s superiority over other state-of-the-art models. Moving forward, this method can be integrated with the High-Efficiency Video Coding (HEVC) standard to restore compression-induced quality degradation during the post-processing stage. This would provide the industry with a practical video restoration solution to significantly improve the visual quality of compressed images.

## Figures and Tables

**Table 1:** Averaged PVD/SD of test sequences for PSNR at QP = 27, 32, and 37.

Method	QP27	QP32	QP37
HEVC	1.07/0.83	1.38/0.82	1.42/0.79
ARCNN	1.07/0.83	1.38/0.82	1.44/0.80
DnCNN	1.06/0.83	1.40/0.83	1.44/0.80
MFQEv2	0.77/0.74	0.98/0.70	0.96/0.67
RFDA	0.63/0.61	0.70/0.63	0.69/0.61
BasicVSR++	—	0.73/0.67	0.71/0.66
STCF	0.57/0.58	0.62/0.59	0.61/0.61
Ours	0.59/0.58	0.57/0.55	0.54/0.53

**Table 2:** Overall performance comparison in terms of $▵\mathrm{PSNR}(\mathrm{dB})/▵\mathrm{SSIM}(\times 10^{-2})$ over the test sequences at four QPs. Video resolutions: Class A (2560 × 1600), Class B (1920 × 1080), Class C (832 × 480), Class D (480 × 240), Class E (1280 × 720). Bold indicates best performance.

QP		Sequences	ARCNN	DNCNN	MFQEv2.0	STDF-R3L	RFDA	BasicVSR++	TVQE	STCF	Ours
			[23]	[24]	[11]	[5]	[12]	[17]	[16]	[28]	LFDM
37	A	Traffic	0.24/0.47	0.24/0.57	0.59/1.02	0.73/1.15	0.80/1.28	0.94/1.52	0.88/1.44	0.91/1.44	**1.04**/**1.64**
		PeopleonStreet	0.35/0.75	0.41/0.82	0.92/1.57	1.25/1.96	1.44/2.22	1.37/2.23	1.49/2.33	**1.62**/**2.43**	1.58/2.37
	B	Kimono	0.22/0.65	0.24/0.75	0.55/1.18	0.85/1.61	1.02/1.86	1.41/2.18	0.99/1.82	1.21/1.94	**1.52**/**2.26**
		ParkScene	0.14/0.38	0.14/0.50	0.46/1.23	0.59/1.47	0.64/1.58	0.86/2.25	0.66/1.76	0.74/1.79	**0.95**/**2.30**
		Cactus	0.19/0.38	0.20/0.48	0.50/1.00	0.77/1.38	0.83/1.49	0.62/1.51	0.85/1.57	**0.93**/**1.61**	0.82/**1.61**
		BQTerrace	0.20/0.28	0.20/0.38	0.40/0.67	0.63/1.06	0.65/1.06	0.71/1.25	0.74/1.34	0.75/1.25	**0.82**/**1.38**
		BasketballDrive	0.23/0.55	0.25/0.58	0.47/0.83	0.75/1.23	0.87/1.40	1.02/1.53	0.85/1.46	**1.09**/1.59	1.06/**1.74**
	C	RaceHorses	0.22/0.43	0.25/0.65	0.39/0.80	0.55/1.35	0.48/1.23	0.76/**1.84**	0.61/1.59	0.69/1.59	**0.86**/**1.84**
		BQMall	0.28/0.68	0.28/0.68	0.62/1.20	0.99/1.80	1.09/1.97	1.17/2.24	1.06/2.02	**1.25**/2.21	1.24/**2.32**
		PartyScene	0.11/0.38	0.13/0.48	0.36/1.18	0.68/1.94	0.66/1.88	0.44/1.71	**0.80**/2.27	0.73/2.28	0.78/**2.36**
		BasketballDril	0.25/0.58	0.33/0.68	0.58/1.20	0.79/1.49	0.88/1.67	0.87/1.67	**0.98**/**2.01**	0.96/1.76	0.89/1.88
	D	RaceHorses	0.27/0.55	0.31/0.73	0.59/1.43	0.83/2.08	0.85/2.11	1.02/2.74	0.86/2.30	1.02/2.47	**1.17**/**2.90**
		BQSquare	0.08/0.08	0.13/0.18	0.34/0.65	0.94/1.25	1.05/1.39	0.61/0.93	**1.25**/**1.74**	1.06/1.48	1.02/1.57
		BlowingBubbles	0.16/0.35	0.18/0.58	0.53/1.70	0.74/2.26	0.78/2.40	0.69/**2.65**	0.83/2.60	0.80/2.53	**0.85**/2.62
		BasketballRass	0.26/0.58	0.31/0.75	0.73/1.55	1.08/2.12	1.13/2.24	1.22/2.66	1.12/2.41	**1.32**/2.63	1.30/**2.73**
	E	FourPeople	0.37/0.50	0.39/0.60	0.73/0.95	0.94/1.17	1.13/1.36	1.13/1.38	1.16/**1.42**	1.11/1.33	**1.20**/**1.42**
		Johnny	0.25/0.10	0.32/0.40	0.60/0.68	0.81/0.88	0.90/0.94	0.99/0.97	**1.12**/**1.33**	1.00/1.13	1.06/1.25
		KristenAndSara	0.41/0.50	0.42/0.60	0.75/0.85	0.97/0.96	1.19/1.15	1.20/1.13	**1.27**/**1.23**	1.12/1.11	1.15/1.21
		Average	0.23/0.45	0.26/0.58	0.56/1.09	0.83/1.51	0.91/1.62	0.95/1.80	0.98/1.82	1.02/1.81	**1.08**/**1.93**
42		Average	0.29/0.96	0.22/0.77	0.59/1.65	0.76/2.04	0.82/2.20	—/—	**0.99**/**2.64**	0.88/2.34	0.97/2.50
32		Average	0.18/0.19	0.26/0.35	0.52/0.68	0.86/1.04	0.87/1.07	0.89/1.25	0.93/1.24	1.07/1.32	**1.09**/**1.55**
27		Average	0.18/0.14	0.27/0.24	0.49/0.42	0.72/0.57	0.82/0.68	—/—	0.87/0.80	**1.05**/0.88	1.03/**1.17**

**Table 3:** The impact of ELPNet’s involvement on PSNR and SSIM within the test sequences.

Method	Fusion Scheme	Δ PSNR	Δ SSIM
Diffusion-only	—	0.78	1.40
ELPNet-only	—	0.51	1.32
Diffusion and ELPNet	Cross-attention	0.96	1.67
Diffusion and ELPNet	Cross-attention and fusion	1.08	1.93

**Table 4:** The effects of DWT and various loss functions on PSNR and SSIM for test sequences. √ indicates that the feature or component was enabled, while × signifies that it was not enabled.

Method	$L_{\mathrm{cha}}$	$L_{\mathrm{ssim}}$	$L_{\mathrm{per}}$	Δ PSNR	Δ SSIM
w/o DWT ELPNet	√	×	×	0.40	0.97
ELPNet	√	×	×	0.48	1.24
ELPNet	√	√	×	0.49	1.29
ELPNet	√	√	√	0.51	1.32

## Data Availability

The data presented in this study are available on request from the corresponding author.
